# Metagenomic insights into the plasma virome of Brazilian patients with prostate cancer

**DOI:** 10.1080/23723556.2023.2188858

**Published:** 2023-03-15

**Authors:** Dalila Luciola Zanette, Karoline Brito Caetano Andrade Coelho, Eneas de Carvalho, Mateus Nobrega Aoki, Jeanine Marie Nardin, Larissa Araújo Lalli, Rafael dos Santos Bezerra, Marta Giovanetti, Victória Simionatto Zucherato, Gabriel Montenegro de Campos, Jardelina de Souza Todão Bernardino, Vincent Louis Viala, Massimo Ciccozzi, Luiz Carlos Junior Alcantara, Sandra Coccuzzo Sampaio, Maria Carolina Elias, Simone Kashima, Dimas Tadeu Covas, Svetoslav Nanev Slavov

**Affiliations:** a Laboratory for Applied Science and Technology in Health, Carlos Chagas Institute, Oswaldo Cruz Foundation (FIOCRUZ), Professor Algacyr Munhoz Mader, Curitiba, Parana, Brazil; b Department of Anatomy and Surgery, Faculty of Medicine of Ribeirão Preto, University of São Paulo, Ribeirao Preto, Sao Paulo, Brazil; c Butantan Institute, São Paulo, São Paulo, Brazil; d Department of Urology, Erasto Gaertner Hospital, Curitiba, Parana, Brazil; e Blood Center of Ribeirão Preto, Faculty of Medicine of Ribeirão Preto, University of Sao Paulo, Ribeirao Preto, Sao Paulo, Brazil; f Department of Science and Technology for Humans and the Environment, University of Campus Bio-Medico di Roma, Rome, Italy; g Laboratory of Flaviviruses, Oswaldo Cruz Foundation, Rio de Janeiro, Rio de Janeiro, Brazil; h Epidemiology and Statistic Unit, University of Campus Bio-Medico di Roma, Rome, Italy

**Keywords:** Virome, prostate cancer, metagenomics, viral diversity, next-generation sequencing

## Abstract

Growing evidence suggests that metavirome changes could be associated increased risk for malignant cell transformation. Considering Viruses have been proposed as factors for prostate cancer induction. The objective of this study was to examine the composition of the plasma metavirome of patients with prostate cancer. Blood samples were obtained from 49 male patients with primary prostate adenocarcinoma. Thirty blood donors were included as a control group. The obtained next-generation sequencing data were analyzed using a bioinformatic pipeline for virus metagenomics. Viral reads with higher abundance were assembled in contigs and analyzed taxonomically. Viral agents of interest were also confirmed by qPCR. Anelloviruses and the Human Pegivirus-1 (HPgV-1) were the most abundant component of plasma metavirome. Clinically important viruses like hepatitis C virus (HCV), cytomegalovirus and human adenovirus type C were also identified. In comparison, the blood donor virome was exclusively composed of torque teno virus types (TTV) types. The performed HPgV-1 and HCV phylogeny revealed that these viruses belong to commonly detected in Brazil genotypes. Our study sheds light on the plasma viral abundance in patients with prostatic cancer. The obtained viral diversity allowed us to separate the patients and controls, probably suggesting that malignant processes may influence virome composition. More complex and multiple approach investigations are necessary to examine the likely causal relationship between metavirome and its nvolvement in prostate cancer.

## Introduction

Prostate cancer is the most common malignancy among male individuals and the second most commonly diagnosed type of cancer, placing this oncologic disease as a leading cause of death worldwide. Recent estimates assign more than a million of newly diagnosed cases and an annual death toll rate of 300,000 cases. Future prognoses are not optimistic as they calculate more than 2 million new cases and ~ 700 thousand deaths in 2040.^1^ Prostate carcinogenesis is a process of high complexity, where chronic intra-prostatic inflammation significantly impacts the malignant transformation of the prostatic tissues in addition to other well-established etiologic factors like older age, African origin and family history.^1,2^ Chronic inflammation, when caused by viral infections, has also been suggested^3^ as a factor contributing to malignant prostatic transformation. Additionally, overexpression of genes, related to inflammation and immune responses including COX-2, RNASEL and TRL4 have been correlated to risk of prostate malignant processes, suggesting that the interplay between infection and immune activation may lead to prostate cancer development.^4^

Persistent viral infections especially caused by herpesviruses (human cytomegalovirus, CMV; Epstein Barr virus, EBV; Human herpesvirus-8, HHV-8) and human papillomaviruses (HPV) have already been suggested as possible factors for malignant transformation of prostate cells. For example, infectious mononucleosis caused by EBV has been correlated to increased PSA levels, observed in patients with prostate cancer.^5^ Additionally, virus-specific proteins involved in viral replication may interact with cellular proteins that control the cell cycle, thus altering the cellular proliferative capacity and participating in the malignant transformation of the prostate tissues.^6^ Another suggested mechanism that is also involved in malignant transformation of the cells includes integration of viral genes into the host genome and its disruption.^7^ Genetic material of different viral agents has also been directly detected in cancerous prostate tissues by molecular methods.^8,9^

Recent surveys are consistent that changes in the virome composition have been tightly related to malignant processes.^10,11^ In several types of oncologic diseases, i.e. gastric, lung, hematologic and gynecological malignancies, the involvement of microbiome changes in relation to these malignant processes has been largely discussed.^12–15^ Indeed, the next-generation sequencing (NGS) and viral metagenomics are the most powerful tools to describe the viral composition and its changes of a wide variety of samples including clinical ones.^16^

To our knowledge, there are no studies which evaluate the blood virome in patients with prostate cancer. In an attempt to characterize the virome composition in plasma samples obtained from patients with prostate cancer, we applied NGS and viral metagenomic analysis to get a deeper insight into the viral abundance of plasma samples from patients with this oncologic disease. We also evaluated by direct molecular detection some characteristics of the most abundant in viral reads infections (genotypes, amplification threshold) that were identified among patients with prostate cancer.

## Materials and methods

### Clinical samples and patients

In this study, we included all patients who were diagnosed with prostate cancer for the period between November 2020 and June 2021 and were naïve to chemotherapy or radiological treatment. Forty-nine blood samples were collected from the same number of patients from the Erasto Gaertner Hospital in the city of Curitiba, state of Parana, Brazil. All the individuals were informed about the study and signed a written informed consent when they agreed to participate. The study was approved ethically under the following number CAEE 36.931.020.91001.0098. Additionally, in this study we included a control group of 30 blood donors, who were all eligible for blood donation. The blood donor samples were obtained between July-September, 2020 from the Blood Center of Ribeirão Preto, São Paulo State, Southeast Brazil, located in a similar geographic area. After blood collection, the plasma was separated from the cellular component by low-speed centrifugation (3,000 rpm for 5 min) and stored under −80ºC until used for the virome analyses.

### Sample pre-preparation, nucleic acids extraction and NGS sequencing

Initially, 600 μL of plasma was pre-treated with Turbo DNAse (ThermoFisher Scientific) for host/bacterial DNA removal. After DNAse inactivation, five individual plasma samples (one pool was prepared with six plasmas) were assembled in pools. The blood donor pools were composed of 10 plasma samples. The pooling of samples was adopted in order to reduce the cost of the sequencing and test all collected samples by viral metagenomics. Nucleic acids were extracted from the total pool volume using the High Pure Viral Nucleic Acid Large Volume Kit (Roche) with minor modifications, i.e. the use of GenElute Linear Polyacrylamide carrier (LPA) (Merck) for nucleic acid concentration and isopropanol for precipitation. After extraction, nucleic acids were recovered in 50 µl of pre-heated to 70°C volume nuclease-free water. Five µl of extracted nucleic acids were submitted to reverse transcription using the Superscript III First-Strand Synthesis System (ThermoFisher Scientific). The amplification of the cDNA was performed using the QuantiTect Whole Transcriptome Kit (QIAGEN). The sequence libraries were prepared using the DNA Prep Library Preparation Kit (Illumina) and Nextera DNA CD Indexes using 500 ng of amplified product for tagmentation. The pair-end sequencing of the dual-indexed libraries was performed by Illumina NextSeq 2000 sequencing platform using the NextSeq P3 flowcell (300 cycles) (Illumina), following the manufacturer’s instructions.

### Bioinformatic processing of the raw sequencing data and taxonomic classification of viral reads

The obtained raw sequence data were submitted to quality control analysis using FastQC v.0.11.8 software. Trimming and adapter removal were performed applying TrimGalore v.0.6.6 and Fastp v.0.23.1 in order to select the best quality reads and free of adapters. For the metagenomic analysis, we used only reads with >30 of quality score. For virome taxonomic classification, we used the Kraken2 v.2.0.8 with the application of the genomic minikraken2 bank. Kraken2 was also used to subtract the human, bacterial and parasitic reads, which were excluded from the further analysis. De novo” assembly was performed using SPAdes v.3.13.0 to generate viral contigs. Finally, to perform taxonomic classification based on protein identity, we applied Blastx as implied by the Diamond v.0.9.29 software. This pipeline was also applied for contaminant and artifact screening which were removed after the BlastX step.

### Analysis of the viral diversity

The abundance of the viral communities was estimated based on a normalized number of read counts obtained during the sequencing. The normalization values of each sample were obtained by the multiplication of the raw read number by a specific factor that was calculated by the division of the number of trimmed reads of the sample with the lowest read number by the trimmed read number of the sample of interest. The analysis was performed using taxa which were represented by up to three normalized reads. The normalized sequence data was analyzed by two forms: using the complete sequencing dataset and a manually curated subset. For manual data curation, viruses that do not cause human infections, phages and artifacts were manually removed from the Kraken2 results. Additionally, the Kraken2 outputs were transformed to biom tables using Kraken-biom and then, in R programming environment, we used the package phyloseq to calculate and plot the Shannon and Simpson indices of diversity; ggplot2, heatmap2 and factorextra packages were also used for plotting data.

### Phylogenetic analysis

Phylogenetic analysis was performed using a dataset of complete genomes which were obtained from the NCBI (National Center for Biotechnology Information, https://www.ncbi.nlm.nih.gov/). The alignment was performed using MAFFT v.7.429 (automatic), and the phylogenetic signal was evaluated using the Tree Puzzle v.5.2 program. To reconstruct the phylogenetic history of the identified viruses, we used the IQ-TREE v.18 applying the approximate maximum likelihood method with a statistical support of ultrafast bootstrap with 1,000 replicates. The phylogenetic tree was visualized and edited in FigTree v.1.4.4 v. software.

### Direct detection of viral pathogens

We performed direct confirmation of two viral pathogens which were identified by metagenomic analysis, i.e. Human Pegivirus-1 (HPgV-1) and the hepatitis C virus (HCV). The detection of HPgV-2 RNA was performed using primers and probes available in the literature.^17^ In brief, in this reaction were applied the forward (5´-GGCGACCGGCCAAAA-3´) and reverse (5´-CTTAAGACCCACCTATAGTGGCTAC-3´) primers in concentrations of 600 nM and 200 nM of the probe (FAM-5´-TGACCGGGATTTACGACCTACCAACCCT-3´-TAMRA) in a final volume of 20 μL. The reaction was performed using GoTaq Probe 1-Step RT Master Mix (Promega) in QuantStudio 6 Flex (ThermoFisher Scientific) cycler under the following conditions: initial step of reverse transcription at 40°C for 40 min, followed by denaturation at 95°C for 2 min and 40 cycles consisting of denaturation at 95°C for 15 min and one step of annealing and extension at 60°C for 1 min. The detection of HCV RNA was performed using the kit NAT HIV/HCV/HBV (BioManguinhos, Rio de Janeiro, Brazil) following the manufacturer’s instructions. The cycling conditions of the reaction were: initial reverse transcription step at 51°C for 30 min, denaturation at 95°C for 10 min and 50 cycles consisting of denaturation at 95°C for 0:30 sec and a combined annealing and elongation step at 60°C for 1 min. The reactions were performed in the same equipment used for the testing of HPgV-1 RNA.

### Serological testing for HCV IgG antibodies

The detection of HCV IgG antibodies was performed using the Architect anti-HCV reagent kit (Abbot) following the manufacturers’ instructions. The testing for anti-HCV IgG antibodies was performed in the Laboratory of Serology, Blood Center of Ribeirão Preto adhering strictly to all measures related to routine HCV antibody detection.

### Statistical analysis

Groups of patients with prostate cancer were compared using the unpaired Kruskal–Wallis test. P-values of <0.05 were considered as significant.

## Results

### Patient demographics and clinics

The average age of the patients included in this study was 70 years (range 49–84 years of age) and all of them declared themselves as Caucasians. Median total of the prostate-specific antigen (PSA) level was 10.25 ng/mL and interquartile range, IQR was 14.86 ng/mL. The mean body mass index (BMI) was 27.1 (IQR 14.9). All patients were diagnosed with primary prostate adenocarcinoma. In five of them, the cancer was metastatic (*n* = 5/49, 10.2%). The tumor grade distribution according to ISUP (International Society of Urological Pathology) classification was: grade 1 *n* = 13 (29.5%); grade 2, *n* = 6 (13.6%); grade 3, *n* = 9 (20.5%); grade 4, *n* = 10 (22.7%) and grade 5, *n* = 6 (13.6%). Kruskal–Wallis tests showed no significant differences in BMI (*p* = .237), PSA (*p* = .199) and age (*p* = .695) among the pools of patients’ samples and, therefore, we assembled them randomly. The control group consisted of blood donors with gender distribution as follows: 60% female and 40% male blood donors with a mean age of 38 years. All of them were negative for the routinely tested blood-borne infections and eligible for blood donation.

### Sequencing characteristics of the analyzed samples

**Figure 1.**
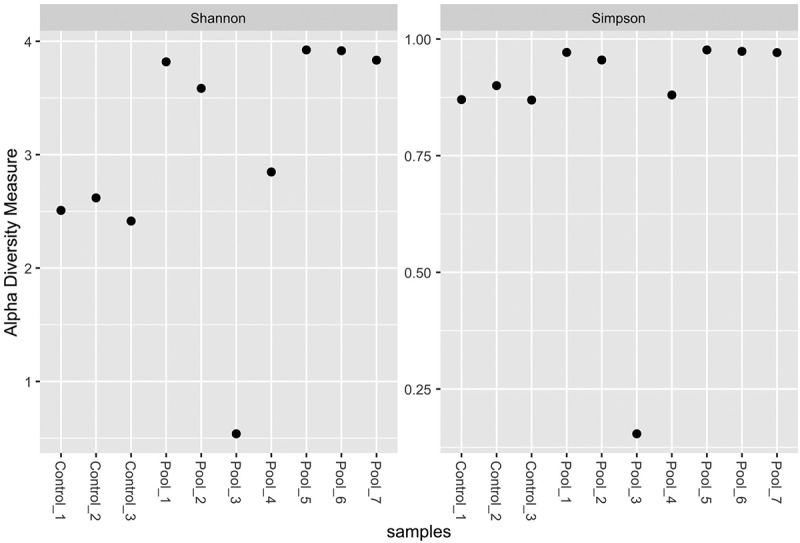
Scatterplot of Shannon and Simpson indexes for Alpha diversity measures of the all classified viral reads in the examined samples. Pool 3 shows very low values in both occasions which is related to the identification of low number of viral taxa, albeit with high read number.

**Figure 2.**
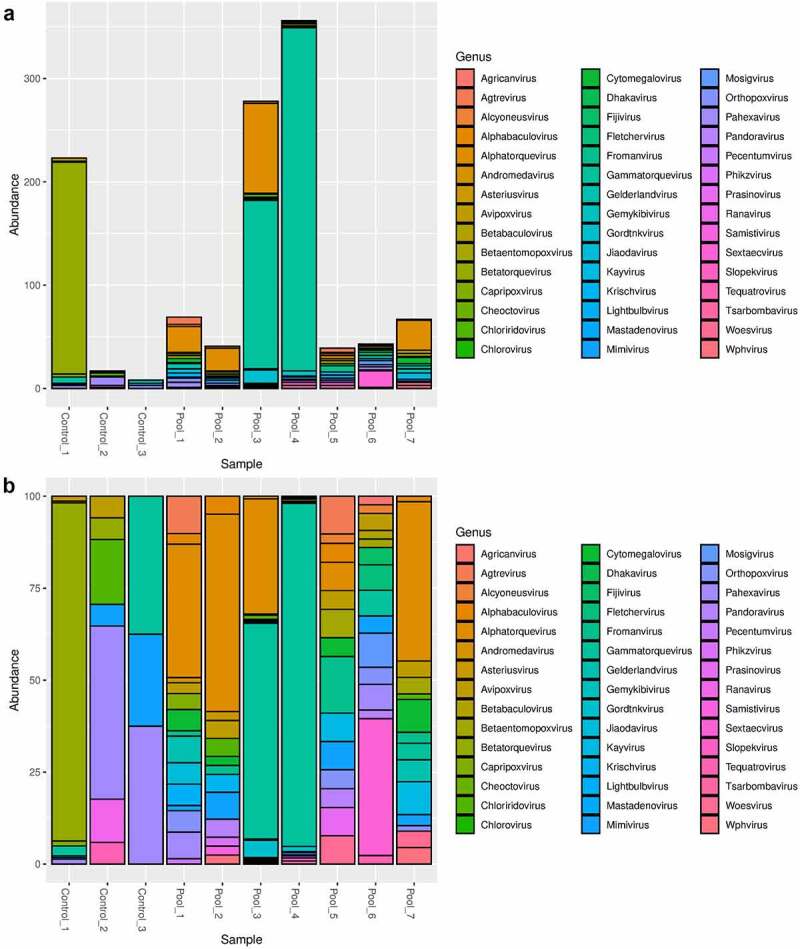
Abundance of each viral genus detected in the non-curated dataset. (a) Relative abundance of the viral genera in the samples. In the X axis, the laboratory number of the pools is given, while in the Y-axis, the percentage of the sum of each identification of genus. Across all pools one can observe the high abundance of anelloviruses. (b) The same data was presented in absolute values.

The performed NGS on pools of patients with prostate cancer generated a median of 84, 062, 683 total reads. The quantitative characteristics of the NGS after trimming and retrieving the bacterial and human reads are shown in Table 1. The median number of viral reads for all pools was 4558 (0.005% of the total number of reads). The controls generated a median of 79,043, 896 reads and as viral reads were classified a median of 159,022 reads (0.2%). Considering the non-curated viral data, we calculated the Shannon and Simpson indexes of alpha abundance (Figure 1). All samples (with the exception of pool 3) were relatively similar in virus abundance level and representativity of the natural occurrence of the identified viral infections among the evaluated population. We observed that both Shannon and Simpson indexes for pool 3 showed lower values related to low abundance levels. In this relation, pool 3 showed the presence of only three predominant viral genera. All other samples had similar values for the Simpson index, and for the Shannon index we observed slightly low levels for the controls. The controls showed a reduced number of virus genera and also read number that could explain the lower values of the Shannon index. The viral general abundance is shown in Figure 2.

**Table 1.** Quantitative representation of the obtained reads from the plasma samples from patients with primary prostatic adenocarcinoma.

Pool number	Total read number	Reads after trimming	Unclassified reads	Human reads	Bacterial reads	Viral reads
Pool 1	48,907,102	47,905,702	13,148,382	34,756,734	36,004	586
Pool 2	88,341,428	86,320,012	27,755,645	58,487,186	77,181	1529
Pool 3	86,608,503	85,432,732	26,841,137	58,458,464	117,301	15,830
Pool 4	73,195,695	72,123,014	23,878,691	48,122,508	116,380	5435
Pool 5	95,344,714	92,567,178	23,916,641	68,577,243	71470	1824
Pool 6	89,458,829	87,236,711	26,915,870	60,219,091	99,841	1909
Pool 7	106,582,509	104,329,431	27,611,692	76,634,095	81,881	1763

### Viral abundance

**Figure 3.**
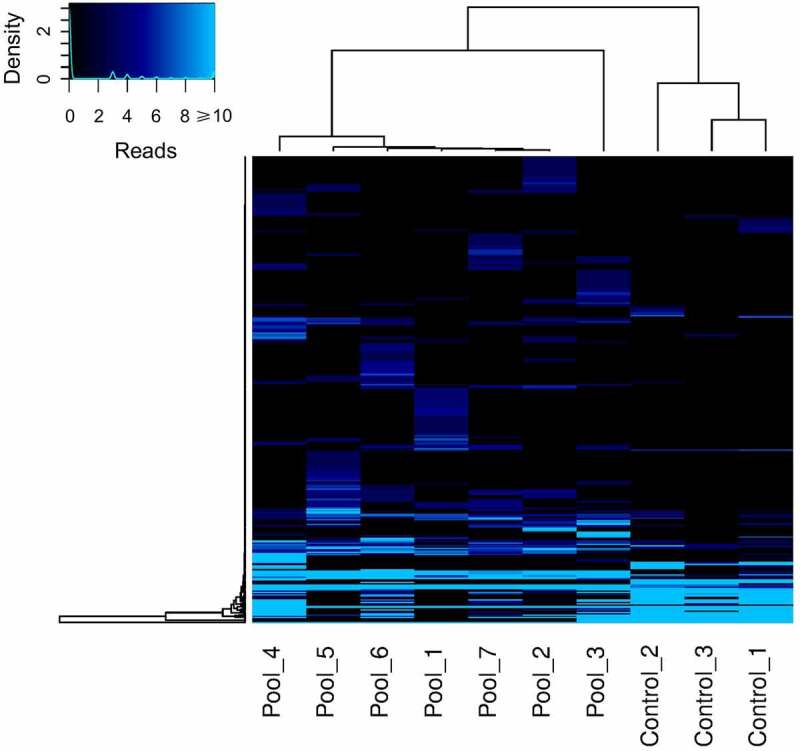
Heatmap showing the viral abundance based on the total number of viral reads obtained from each analyzed sample. The keycode (upper left) indicates the correlation between the color scheme and the number of reads. A hierarchical cluster is presented for both samples and viral species/genus. The clustering between the group of patients and controls can also be observed.

**Figure 4.**
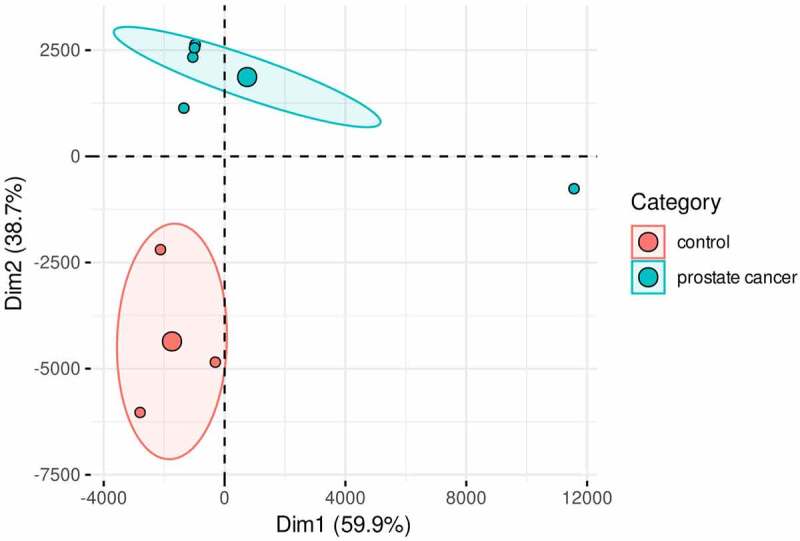
Principal component analysis (PCA) showing that patients and control group were clustered separately. The small circles represent the individual samples, while the big circles are centroid for each group. The ellipse englobes 95% of confidence interval. The sum of PCA1 and PCA2 is close to 100% that means that by this way can be explained almost all dataset variation.

**Figure 5.**
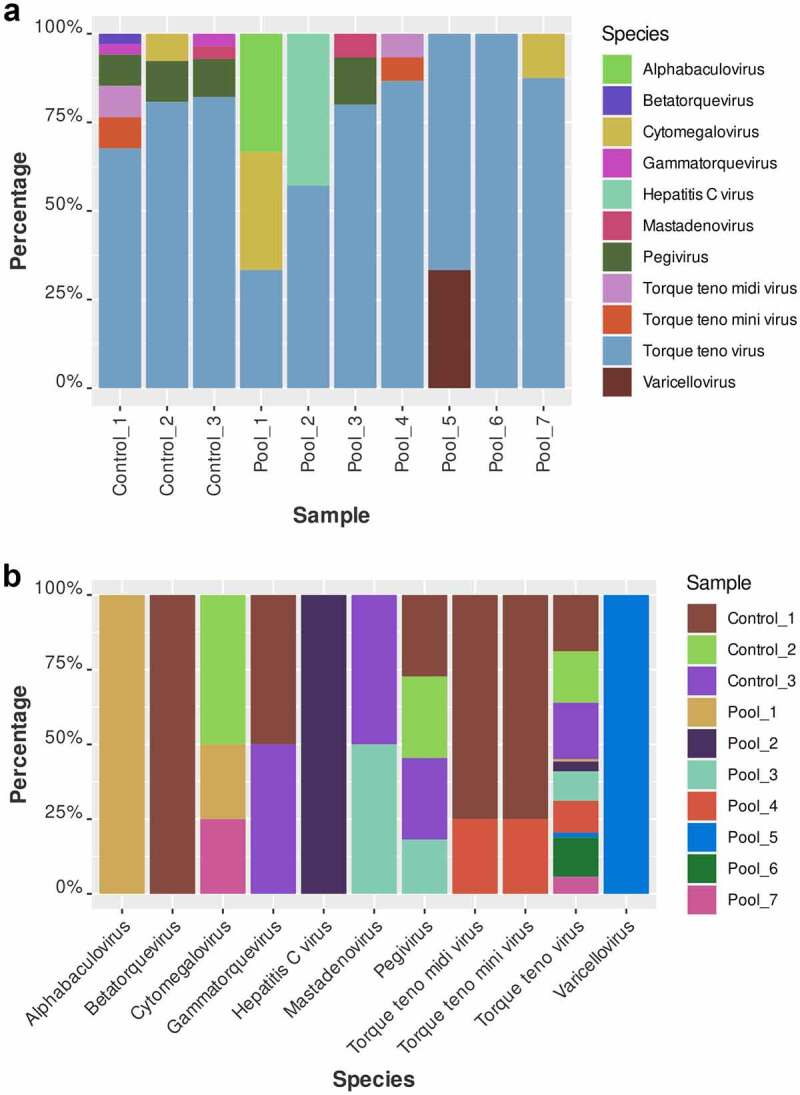
Abundance of human viruses in patients and controls (curated dataset). (a) Relative abundance of the viral reads in each pool. In the X axis the laboratory number of the pools is given, while in the Y-axis, the percentage. In pool 2 can be observed the reads obtained from HCV while in pool 3 HPgV-1 was detected. In pool 3 we were also able to detect low number of reads belonging to human adenovirus type C. Across all pools one can observe the high abundance of anelloviruses. (b) Relative abundance of the viral species per pool. On the X-axis are represented the viral species.

The obtained viral abundance by species is shown by a heatmap in Figure 3. This data allowed us to cluster the patients and the controls as separate groups. In addition to this, we build a principal component analysis (PCA) plot that also showed that each formed group has a similar variability pattern (Figure 4). In order to examine in more detail the human viruses among patients with prostate cancer, we curated the total abundance virome. In Figure 5 is shown the curated virome of patients with prostate cancer and controls containing viruses that cause human infection. It showed the extensive presence of commensal viruses (mainly anelloviurses and HPgV-1) (Figure 5 a,b). Indeed, the most abundant anelloviruses throughout all pools were the torque teno viruses (TTV) with the most abundant species TTV-15, 16 and especially TTV-22. HPgV-1 was another commensal virus detected with expressive read abundance in pool 3 (Figure 5a, b). The high HPgV-1 read abundance of allowed the assembly of complete viral genome. Interestingly, we were also able to identify Torque teno midi virus 9 (TTMDV-9) in pool 4 (Figure 5a, b) which is also a commensal anellovirus but diverges from the TTV group. Importantly, human pathogenic viral agents were also detected in patients with prostate cancer. In pool 2 (Figure 5a) we detected a number of HCV reads which were further assembled in 422 bp contig which was phylogenetically characterized. We also detected a low number of CMV reads in pools 1 and 7 and reads characterized as human adenovirus type C in pool 3 (Figure 5 a, b). In the controls, we detected exclusively the presence of commensal viruses and will low abundance as shown in Figure 5. The most abundant viruses among blood donors were TTV of the types 10, 12, 16 and 22 as well as TTMV-1 and 5.

### Phylogenetic analysis

**Figure 6.**
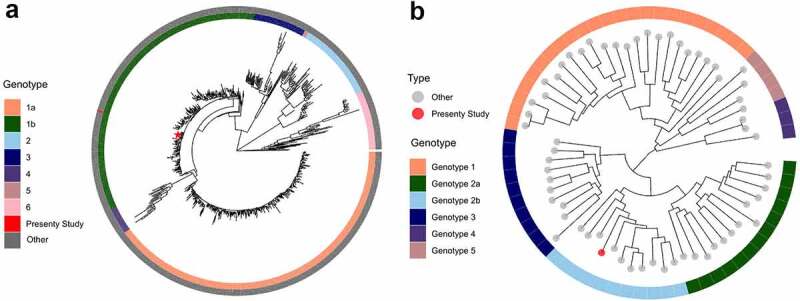
Genotype classification of the hepatitis C virus (HCV) and Human Pegivirus-1 (HPgV-1) isolates obtained via metagenomic analysis from patients with prostatic cancer by phylogenetic analysis. (a) The reconstructed Maximum Likelihood tree demonstrated that HCV strain belonged to subgenotype 1b and clustered together with other strains obtained from Brazil and worldwide. (b) Maximum Likelihood tree obtained from the complete HPgV-1 genome from patient with prostatic cancer, was consistent with the identification genotype 2 (subgenotype 2B) which is the predominant genotype worldwide.

From the generated HCV and HPgV-1 contigs obtained from patients with prostate cancer, we reconstructed phylogenetic trees. The HCV phylogenetic analysis with a representative dataset containing 867 reference genomes showed that it belongs to the 1b subgenotype (Figure 6a). We also reconstructed the phylogenetic history of HPgV-1 using a dataset of 66 reference genomes of the genotypes 1 to 5. Our results demonstrated that HPgV-1 was classified as genotype 2, subgenotype 2b, which is the most prevalent worldwide (Figure 6b).

### Molecular confirmation of HCV and HPgV-1 RNA in individual samples

**Figure 7.**
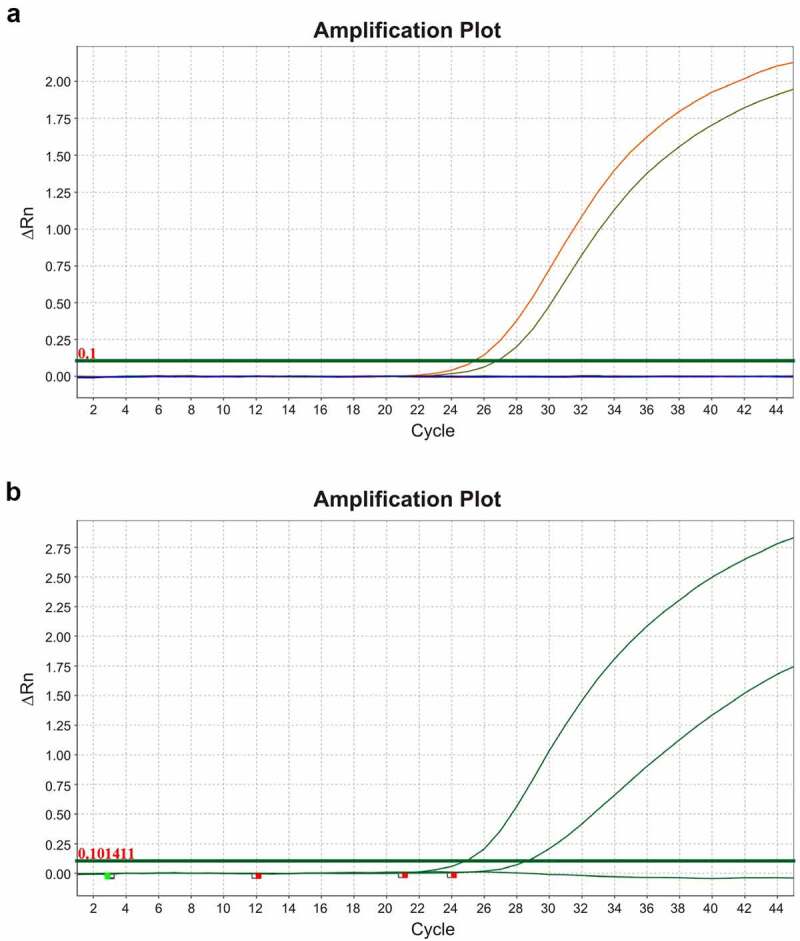
Amplification plots of the positive samples for Human pegivirus-1 (HPgV-1) and hepatitis C virus (HCV) obtained from patients with prostate cancer. (a) Amplification plot of the positive for HPgV-1 sample. In orange is presented the amplification curve of the clinical sample (Ct = 25.3) and in green the positive HPgV-1 control (Ct = 26.7). (b) Amplification plot of the positive for HCV RNA clinical sample obtained from patient with prostate cancer. Cycle threshold of the clinical sample is 24.9 and of the positive control is 28.6.

We additionally performed a direct molecular confirmation of HPgV-1 and HCV RNA in the individual plasma samples which composed each pool of patients with prostate cancer. In the pool with a high number of HPgV-1 reads (pool 3), HPgV-1 RNA was detected in only one patient with 75 years of age and PSA values 49 ng/mL (normal values for patients between 70 and 79 years between 0.0 and 5.5). The HPgV-1 infection showed Ct = 25.3 (threshold = 0.1) that was probably related to high viral load. Furthermore, HCV detection was performed both serologically and molecularly. The HCV-positive patient was an older individual of 70 years of age and PSA value of 3.72 ng/mL. The serological anti-HCV IgG detection showed a high optical density (cutoff value 0.760; sample optical density >3000). The detection of HCV RNA showed a Ct = 24.9 (threshold = 0.1), probably characterizing a chronic HCV infection related to high viral load. The amplification plots of HPgV-1 and HCV are shown in Figure 7.

## Discussion

In this study, we characterized the plasma virome composition of primary adenocarcinoma patients with different ISUP grades. We assumed that the plasma virome of patients with prostatic cancer may show characteristic abundance that might be reflection of the malignant prostatic disease. Additionally, viruses have been largely implicated as cofactors that might unlock pathologic events leading to prostate cancer. For this reason, using of deep metagenomics for characterization of the metavirome composition of different clinical specimens including plasma of such patients is of particular interest.

The viral diversity of the examined samples was similar, although the controls showed slightly reduced variability considering the Shannon diversity. Indeed, in the heatmap plot, we were able to cluster separately the controls and the patients indicating that each group has specific viral composition. In addition, the performed PCA analysis reinforced the specific viral pattern in patients and controls.

Concerning, the curated viral data, the plasma metavirome of patients with prostate cancer revealed the presence of a variety of viral agents. As expected, the highest abundance was attributed to anelloviruses, predominantly TTV and at lesser frequency TTMDV (across the samples, TTV-15,16 and 22 as well as TTMDV-9 were the most abundant). The role of the anelloviruses as pathogenic agents has been debated. These viruses constitute a normal part of the human virome and are acquired early in life without causing an apparent disease.^18^ However, elevated TTV loads have been observed in patients with different malignancies^19–21^ including prostate cancer^22^ probably as a result of immunological dysregulations predisposing to bloom in the annelovirus diversity.^23,24^ In blood donors, we exclusively detected a variability of TTV viruses but with lower read abundance compared to patients with prostate cancer. The presence of anelloviruses types in plasma of patients with prostate cancer requires more detailed investigations especially related to estimation of viral loads and TTV diversity.

HPgV-1, another commensal virus, was almost exclusively detected among patients with prostate cancer. In one of the patients, the infection was presented with high viral load that permitted detailed genomic investigation of the infecting strain. In that particular case, the HPgV-1 read number and load might be related to immunological alterations caused by cancer itself, and the older age of the patient as observed in other studies.^25^ The HPgV-1 phylogenetic analysis showed that it belongs to genotype 2 (subgenotype 2B) which is widely distributed in Brazil, Europe and the USA.^26–28^ Commensal viruses make an indispensable part of the normal human metavirome and it is difficult to make any conclusions about their participation in prostate carcinogenesis. Nevertheless, recent studies suggest that the level of commensal virus replication and read number might be useful as a marker to evaluate the immunological competence of the patients.^29,30^

HCV reads were also detected in one of the samples permitting performing of phylogenetic analysis. HCV is an oncogenic virus, but its involvement in extrahepatic cancers has been a matter of debate. Although most studies describe an inverse relationship between HCV infection and prostate cancer,^31^ extrahepatic malignancies like gallbladder, bile duct, ovarian cancers and non-Hodgkin lymphoma^32^ have been attributed to HCV. We believe that HCV identification in our case is a coincidental finding, and we did not have clinical information as to link the obtained results to when and how the infection was acquired. Nevertheless, in this particular case, the viral metagenomics was crucial to detect HCV RNA in this patient and help for his clinical management, as up to this study, the patient´s HCV status was unknown. This reinforces the clinical utility of viral metagenomics for the detection of unsuspected infections. More detailed investigations of the HCV parameters showed elevated load and high antibody titer revealing untreated, chronic course infection. Additionally, the phylogenetic analysis showed HCV subgenotype 1b which is a common Brazilian subgenotype.^33^ More studies are necessary to establish a possible causal relationship between HCV infection and extrahepatic types of cancer. We also identified a low number of CMV and human adenovirus type C reads but due to their frequent detection in states of immune suppression, we believe that it can be regarded as a coincidental finding.

In conclusion, our results provided insights regarding the plasma virome composition among patients with prostate cancer. The obtained viral diversity allowed well-defined separation of the patients and the controls, suggesting that viral diversity and abundance might be the result of the immunological changes induced by the malignant processes. More studies appear to be necessary in order to evaluate its role of the plasma virome and viruses on the malignant transformation of the prostatic cells. Nevertheless, our study gives a preliminary overview on the plasma virome of patients with prostate cancer, its composition and diversity.

## Data Availability

The assembled viral sequences have been deposited in the NCBI GenBank under the following accession numbers: OM982648, OM982649 (https://www.ncbi.nlm.nih.gov/nuccore/OM982648 and https://www.ncbi.nlm.nih.gov/nuccore/OM982649).

## References

[1] Culp MB, Soerjomataram I, Ja E, Bray F, Jemal A. Recent global patterns in prostate cancer incidence and mortality rates. Eur Urol. 2020;77(1):38–9. doi:10.1016/j.eururo.2019.08.005. 31493960

[2] de Bono JS, Guo C, Gurel B, De Marzo AM, Sfanos KS, de Bono JS, Mani RS, Gil J, Drake CG, Alimonti A. Prostate carcinogenesis: inflammatory storms. Nat Rev Cancer. 2020;20(8):455–469. doi:10.1038/s41568-020-0267-9. 32546840

[3] Sfanos KS, Sauvageot J, Fedor HL, Dick JD, De Marzo AM, Isaacs WB. A molecular analysis of prokaryotic and viral DNA sequences in prostate tissue from patients with prostate cancer indicates the presence of multiple and diverse microorganisms. Prostate. 2008;68(3):306–320. doi:10.1002/pros.20680. 18163428

[4] Yow MA, Tabrizi SN, Severi G, Bolton DM, Pedersen J, Longano A, Garland SM, Southey MC, Giles GG. Detection of infectious organisms in archival prostate cancer tissues. BMC Cancer. 2014;14:579. doi:10.1186/1471-2407-14-579. 25106851 PMC4132904

[5] Sutcliffe S, Nevin RL, Pakpahan R, Elliott DJ, Langston ME, De Marzo AM, Gaydos CA, Isaacs WB, Nelson WG, Sokoll LJ, et al. Infectious mononucleosis, other infections and prostate-specific antigen concentration as a marker of prostate involvement during infection. Int J Cancer. 2016;138(9):2221–2230. doi:10.1002/ijc.29966. 26678984

[6] Kori M, Arga KY. Pathways involved in viral oncogenesis: new perspectives from virus-host protein interactomics. Biochim Biophys Acta Mol Basis Dis. 2020;1866(10):165885. doi:10.1016/j.bbadis.2020.165885. 32574835

[7] Borchmann S. An atlas of the tissue and blood metagenome in cancer reveals novel links between bacteria, viruses and cancer. Microbiome. 2020;9(1):94. doi:10.1186/s40168-021-01039-4. PMC8063312 33888160

[8] Whitaker NJ, Glenn WK, Sahrudin A, Orde MM, Delprado W, Lawson JS. Human papillomavirus and Epstein Barr virus in prostate cancer: koilocytes indicate potential oncogenic influences of human papillomavirus in prostate cancer. Prostate. 2013;73(3):236–241. doi:10.1002/pros.22562. 22851253

[9] Ala-Almohadesin A, Mohammadbeygi M, Bahavar A, Mohammadi MA, Mohamadzadeh N, Abolhasani M. Molecular detection of pathogens causing sexually transmissible infections in patients with prostate cancer and Hyperplasia by quantitative TaqMan real-time PCR assay. Clin Lab. 2019;65(7). doi:10.7754/Clin.Lab.2019.181243. 31307183

[10] Broecker F, Moelling K. The roles of the Virome in cancer. Microorganisms. 2021;9:2538. doi:10.3390/microorganisms9122538. 34946139 PMC8706120

[11] Nakatsu G, Zhou H, Wkk W, Wong SH, Coker OO, Dai Z, Li X, Szeto C-H, Sugimura N, Lam TYT, et al. Alterations in enteric Virome are associated with colorectal cancer and survival outcomes. Gastroenterology. 2018;155(2):529–541. doi:10.1053/j.gastro.2018.04.018. 29689266

[12] Marongiu L, Landry JJM, Rausch T, Abba ML, Delecluse S, Delecluse HJ, Allgayer H. Metagenomic analysis of primary colorectal carcinomas and their metastases identifies potential microbial risk factors. Mol Oncol. 2021;15(12):3363–3384. doi:10.1002/1878-0261.13070. 34328665 PMC8637581

[13] Engel K, Wieland L, Krüger A, Volkmer I, Cynis H, Emmer A, Staege MS. Identification of differentially expressed human endogenous retrovirus families in human Leukemia and Lymphoma cell lines and stem cells. Front Oncol. 2021;11:637981. doi:10.3389/fonc.2021.637981. 33996550 PMC8117144

[14] Gonzalez-Bosquet J, Pedra-Nobre S, Devor EJ, Thiel KW, Goodheart MJ, Bender DP, Leslie KK. Bacterial, Archaea, and viral transcripts (BAVT) expression in gynecological cancers and correlation with regulatory regions of the genome. Cancers (Basel). 2021;13(5):1109. doi:10.3390/cancers13051109. 33807612 PMC7961894

[15] Cai HZ, Zhang H, Yang J, Zeng J, Wang H. Preliminary assessment of viral metagenome from cancer tissue and blood from patients with lung adenocarcinoma. J Med Virol. 2021;93(8):5126–5133. doi:10.1002/jmv.26887. 33595122

[16] Chiu CY, Miller SA. Clinical metagenomics. Nat Rev Genet. 2019;20(6):341–355. doi:10.1038/s41576-019-0113-7. 30918369 PMC6858796

[17] Kriesel JD, Hobbs MR, Jones BB, Milash B, Nagra RM, Fischer KF, Feng Y. Deep sequencing for the detection of virus-like sequences in the brains of patients with multiple sclerosis: detection of GBV-C in human brain. PLoS One. 2012;7(3):e31886. doi:10.1371/journal.pone.0031886. 22412845 PMC3297595

[18] Freer G, Maggi F, Pifferi M, Di Cicco ME, Peroni DG, Pistello M. The Virome and its major component, Anellovirus, a convoluted system molding human immune defenses and Possibly affecting the development of Asthma and respiratory diseases in childhood. Front Microbiol. 2018;9:686. doi:10.3389/fmicb.2018.00686. 29692764 PMC5902699

[19] Tokita H, Murai S, Kamitsukasa H, Yagura M, Harada H, Takahashi M, Okamoto H. High TT virus load as an independent factor associated with the occurrence of hepatocellular carcinoma among patients with hepatitis C virus-related chronic liver disease. J Med Virol. 2002;67(4):501–509. doi:10.1002/jmv.10129. 12115995

[20] Bando M, Takahashi M, Ohno S, Hosono T, Hironaka M, Okamoto H, Sugiyama Y. Torque teno virus DNA titre elevated in idiopathic pulmonary fibrosis with primary lung cancer. Respirology. 2008;13(2):263–269. doi:10.1111/j.1440-1843.2007.01217.x. 18339026

[21] Stefani D, Hegedues B, Collaud S, Zaatar M, Ploenes T, Valdivia D, Elsner C, Bleekmann B, Widera M, Dittmer U, et al. Torque Teno Virus load in lung cancer patients correlates with age but not with tumor stage. PLoS One. 2021;16(6):e0252304. doi:10.1371/journal.pone.0252304. 34077485 PMC8171866

[22] Zhong S, Yeo W, Tang MW, Lin XR, Mo F, Ho WM, Hui P, Johnson PJ. Gross elevation of TT virus genome load in the peripheral blood mononuclear cells of cancer patients. Ann N Y Acad Sci. 2001;945(1):84–92. doi:10.1111/j.1749-6632.2001.tb03868.x. 11708500

[23] Görzer I, Jaksch P, Kundi M, Seitz T, Klepetko W, Puchhammer-Stöckl E, Schildgen O. Pre-transplant plasma Torque Teno virus load and increase dynamics after lung transplantation. PLoS One. 2015;10(4):e0122975. doi:10.1371/journal.pone.0122975. 25894323 PMC4404260

[24] Zanella MC, Cordey S, Laubscher F, Docquier M, Vieille G, Van Delden C, Braunersreuther V, Ta MK, Lobrinus JA, Masouridi-Levrat S, et al. Unmasking viral sequences by metagenomic next-generation sequencing in adult human blood samples during steroid-refractory/dependent graft-versus-host disease. Microbiome. 2021;9(1):28. doi:10.1186/s40168-020-00953-3. 33487167 PMC7831233

[25] Lampe E, Saback FL, Viazov S, Roggendorf M, Niel C. Age-specific prevalence and genetic diversity of GBV-C/hepatitis G virus in Brazil. J Med Virol. 1998;56(1):39–43. doi:10.1002/(SICI)1096-9071(199809)56:1<39:AID-JMV7>3.0.CO;2-O. 9700631

[26] Muerhoff AS, Tillmann HL, Manns MP, Dawson GJ, Desai SM. GB virus C genotype determination in GB virus-C/HIV co-infected individuals. J Med Virol. 2003;70(1):141–149. doi:10.1002/jmv.10375. 12629656

[27] Jõgeda EL, Huik K, Pauskar M, Kallas E, Karki T, Des Jarlais D, Uusküla A, Lutsar I, Avi R. Prevalence and genotypes of GBV-C and its associations with HIV infection among persons who inject drugs in Eastern Europe. J Med Virol. 2017;89(4):632–638. doi:10.1002/jmv.24683. 27603233

[28] Silva ASN, Silva CP, Barata RR, da Silva PVR, Monteiro PDJ, Lamarão L, Burbano RMR, Nunes MRT, de Lima PDL. Human pegivirus (HPgV, GBV-C) RNA in volunteer blood donors from a public hemotherapy service in Northern Brazil. Virol J. 2020;17(1):153. doi:10.1186/s12985-020-01427-6. 33054824 PMC7556973

[29] De Vlaminck I, Khush KK, Strehl C, Kohli B, Luikart H, Neff NF, Okamoto J, Snyder T, Cornfield D, Nicolls M, et al. Temporal response of the human virome to immunosuppression and antiviral therapy. Cell. 2013;155(5):1178–11787. doi:10.1016/j.cell.2013.10.034. 24267896 PMC4098717

[30] Blatter JA, Sweet SC, Conrad C, Danziger-Isakov LA, Faro A, Goldfarb SB, Hayes D, Melicoff E, Schecter M, Storch G, et al. Anellovirus loads are associated with outcomes in pediatric lung transplantation. Pediatr Transplant. 2018;22(1):e13069. doi:10.1111/petr.13069. PMC5811341 29082660

[31] Liu X, Chen Y, Wang Y, Dong X, Wang J, Tang J, Sundquist K, Sundquist J, Ji J. Cancer risk in patients with hepatitis C virus infection: a population-based study in Sweden. Cancer Med. 2017;6(5):1135–1140. doi:10.1002/cam4.988. 28374973 PMC5527979

[32] Kamiza AB, Su FH, Wang WC, Sung FC, Chang SN, Yeh CC. Chronic hepatitis infection is associated with extrahepatic cancer development: a nationwide population-based study in Taiwan. BMC Cancer. 2021;16(1):861. doi:10.1186/s12885-016-2918-5. PMC5100218 27821099

[33] Oliveira ML, Bastos FI, Sabino RR, Paetzold U, Schreier E, Pauli G, Yoshida CF. Distribution of HCV genotypes among different exposure categories in Brazil. Braz J Med Biol Res. 1999;32(3):279–282. doi:10.1590/S0100-879X1999000300005. 10347784

